# Improved dietary intake and readiness among United States Navy Future Sailors following mobile health application use

**DOI:** 10.3389/fdgth.2026.1794165

**Published:** 2026-06-23

**Authors:** Lynn A. Cialdella-Kam, Michael S. Stone, Trisha M. Sterringer

**Affiliations:** 1 Food Utilization and Energy Laboratory, Warfighter Performance Department, Naval Health Research Center, San Diego, CA, United States; 2 Leidos, Inc., San Diego, CA, United States

**Keywords:** attrition, delayed entry program, digital health, Future Sailors, initial military training, mHealth, navy

## Abstract

**Introduction:**

Military recruitment and retention efforts are significantly challenging due to a limited pool of qualified candidates and increasing competition from private industries. Before attending Navy basic training, all non-prior service enlistees enter the Navy's Delayed Entry Program (DEP), which is designed to help recruiters prepare Future Sailors (FS) for military life. Mobile health (mHealth) applications (apps), such as CoachMePlus (CM+), may assist recruiters in enhancing FS readiness. The purpose of this study was to evaluate the effectiveness, usability, and impact of an mHealth app on FS readiness.

**Methods:**

A total of 92 FS (13 females, 79 males; ages = 18–28 years) were enrolled in a training program delivered via the mHealth app CM+. The program consisted of three modules: general military training (GMT), nutrition, and physical training. At baseline, FS completed a survey that included demographics and health history. Body weight and body fat percentage were measured and tracked monthly by recruiters. FS also completed weekly check-ins on the app and logged dietary intakes for 3 days each month, which were reviewed by a registered dietitian nutritionist.

**Results:**

FS logged into the app an average of 2.1 ± 0.2 times per day, and 92.4% (*n* = 85) successfully graduated from basic training. During DEP, knowledge improvements were realized for GMT and nutrition, as evidenced by test score increases: GMT scores improved from =51.7 ± 1.6 to 67.9 ± 2.9, and nutrition scores increased from 58.1 ± 1.1 to 68.8 ± 2.0, (*p* < 0.001). Approximately 64% (*n* = 59) of the FS recorded dietary intake for at least 7 days, reporting lower fiber and higher fat and sodium intake than recommended. Calcium and vitamin D intakes were also insufficient. Self-efficacy for diet and exercise was rated as “rather certain” or “very certain” by over 80% of the participants. FS who completed basic training achieved a higher Recruit Division Commander assessment (*p* < 0.001). Recruiters highlighted real-time tracking and progress feedback as key benefits of the app in qualitative feedback.

**Conclusion:**

CM+ proved to be an effective tool for monitoring real-time achievements during DEP training, improving military-related outcomes and nutrition understanding.

## Introduction

The recruitment and retention of military personnel has become increasingly challenging across all service branches. Currently, fewer than one-third of individuals aged 17–24 years meet eligibility criteria for the armed forces due to factors such as criminal records, obesity, and insufficient education level (i.e., high school diploma or equivalent) (1, 2). These challenges are further compounded by societal trends, including increased competition from the private sector and waning interest in military service among younger generations (3). Between fiscal years 2016 and 2020, 21% of Navy sailors were discharged within the first 3 years of service (early discharge) primarily due to adverse attrition factors, such as failure to meet weight standards (4), low physical fitness (5), alcohol use, and/or smoking (6). Each early discharge is estimated to cost∼$75,000 per enlistee (7), a cost that could be mitigated through improved onboarding and sustained engagement. With a shrinking pool of qualified candidates, retention of service members is critical to maintain a robust and capable military force.

The Navy's Delayed Entry Program (DEP) is designed to support non-prior service enlistees in the transition from civilian life to military service. The DEP achieves this by providing military education, physical training (PT), mentorship, and emotional support. Recruiters serve as the primary point of contact during the DEP, ensuring that Future sailors (FS) are adequately prepared for basic training (8). FS training is delivered through the 2018 Standards, Transitions, Acknowledgement, Requirements, Training (START) Guide, which includes general military training (GMT) and PT instruction (9). Participation in DEP training is encouraged but not mandatory. Nonetheless, significant improvements in the health habits of service members are needed, given a reported 73% rise in overweight/obesity among active duty personnel, with an estimated obesity prevalence of 17.4% (5, 10, 11). The promotion of healthy dietary behaviors can improve weight management, and nutrition education has been shown to enhance diet quality, which is associated with better dietary choices in certain military populations (11). A clearer understanding of the benefits of PT, combined with more effective implementation of structured PT programs, is essential for reducing injuries and lowering attrition rates among military personnel (12). To address these challenges, innovative approaches are required to increase engagement and foster both health and behavioral readiness among recruits.

Mobile health (mHealth) applications (apps) have the potential to enhance the delivery of DEP training and improve readiness for basic training. These apps offer customizable and scalable coaching programs, along with tools for tracking individual and group progress. The acceptability of an mHealth app among young adults is high, with Millennials (born 1981–1996) and Generation Z (born 1997–2012) accounting for ∼75% of all mHealth users (13). mHealth technologies have been designed to promote healthy nutrition behaviors (14–16), improve physical fitness (17–19), optimize body composition (20, 21), and, in military populations, address behavioral disorders (22, 23) and musculoskeletal injuries (24). For FS, mHealth apps can be tailored to deliver GMT, nutrition education and coaching, and PT modules. In addition, these apps can streamline communication efforts by providing recruiters with a centralized platform to monitor and engage with FS. Given their versatility and potential benefits, further evaluation of mHealth apps within the DEP is recommended.

This study is the first, to our knowledge, to implement an mHealth intervention in FS to deliver DEP training. The purpose of this research was to evaluate the impact of an mHealth app, CoachMePlus (CM+), on the physical and behavioral readiness of FS. CM+ was specifically tailored to provide DEP-focused GMT, nutrition education, and PT to FS at Navy Recruiting Command (NRC), Navy Talent Acquisition Group (NTAG) Southwest in San Diego, California. We hypothesized that GMT, nutrition knowledge, and dietary intake would improve following CM+ use, and that military-relevant measures of training effectiveness, including the Recruit Division Commander (RDC) assessment, rate of advancement, DEP ratings, and graduation from basic training, would improve compared with historical norms.

## Methods

In the Navy DEP, CM+ was piloted in a single-arm intervention study, to deliver GMT, nutrition education, and PT in NTAG Southwest between June 2021 and January 2022. The study protocol was approved by the Naval Health Research Center Institutional Review Board (protocol number NHRC.2021.0010) in compliance with all applicable federal regulations governing the protection of human subjects.

### Recruitment and baseline data

FS were recruited during the first month of DEP. Written informed consent was provided by all participants prior to baseline data collection. Upon enrollment, body weight (BW, kg) and body fat percentage (BF%) were measured using a bioelectrical impedance analysis scale (BF-350 Total Body Composition Analyzer; Tanita Corporation, Arlington Heights, IL). FS were then assigned a CM+ account, which they used to complete a baseline survey that included demographics, dietary and exercise patterns and behaviors, and chronic disease health history. The baseline survey was a combination of investigator designed questions to ascertain background information as well as validated questionnaires on health behaviors (25).

### Intervention

#### Initial 10-week program

The training delivered through CM+ consisted of an initial 10-week program to parallel the standard DEP training plan and included three modules: GMT, nutrition education, and PT. The GMT and PT modules were based on the 2018 START Guide. The 2019 Navy Operational Fitness and Fueling System (NOFFS) was used to develop the nutrition education modules and included the following topics: Fundamentals of Fueling, Nutrition Rules to Live By, Energy Balance, Portion Sizes, Eat the Rainbow, Hydration, Fueling for Physical Training, Macronutrients, and Recovery Nutrition. FS had access to this information on the NOFFS; however; in the app the information was housed in one location, enhanced with tracking, better visuals, and communication features. FS could review educational content on their phone, track training progress, view video examples/instruction of PT, log dietary intake, and communicate with their recruiter. At the beginning of each week, the FS received a list of tasks and were required to complete a weekly check-in questionnaire. Short assessments were delivered via the app weekly for the GMT module and biweekly for the nutrition module. At the monthly DEP meeting, the research team met with FS to obtain app feedback and recruiters were asked to enter BW and BF% for each FS.

### Post 10-week program

For the post 10-week program, the content delivery was customized for each FS. Specifically, any GMT or nutrition content was reassigned to FS if they performed unsatisfactorily on knowledge checks. For PT, FS were assigned to the appropriate level of the 2019 NOFFS Large Deck Series training based on their training status. This training is designed to better prepare FS for service on Navy vessels focusing on functional movements that may replicate daily shipboard tasks.

### Delayed entry program and basic training outcomes

Basic training outcomes and historical reference ranges were provided by NRC leadership and included the RDC assessment, rate of advancement, DEP ratings, and AFQT scores. The RDC assessment is a modified version of the Navy's Physical Readiness Test and consists of a 1.5-mile run, push-ups, and a plank hold (26). FS receive a score between 0 and 100 for each event with a maximum score of 300, and results are reported as a percentage out of 100. The AFQT score is derived from subtests of the Armed Services Vocational Aptitude Battery (ASVAB), including arithmetic reasoning, mathematics knowledge, paragraph comprehension, and world knowledge (27). AFQT scores are reported as percentiles between 1 and 99. Rate of advancement and DEP ratings refer to the test that recruits take when they first get to boot camp. Individuals who score above a certain threshold are advanced to the next pay grade. Graduation from basic training was also provided.

### System usage data and qualitative feedback

Objective system usage data were collected to estimate participant engagement with the CM+ app. The data included were the total number of times the CM+ app was accessed during the study period and the average number of times the CM+ app was accessed per day. Recruiters provided qualitative feedback regarding the CM+ app.

### Dietary intake

Participants logged diet records (i.e., all foods, beverages, dietary supplements) for 3 days (2 weekdays, 1 weekend day) prior to each monthly DEP meeting. Total energy, macronutrient, and micronutrient intakes were quantified by the CM+ app from the foods recorded. Absolute intake and intake relative to total energy and BW (pre-DEP) were also reported. Macronutrient intake from FS were compared with reference ranges from the Academy of Nutrition and Dietetics, Dietitians of Canada, and the American College of Sports Medicine nutrition and athletic performance guidelines (28). Vitamin and mineral intakes were considered sufficient if intake was equal to or greater than the estimated average requirement (level sufficient to meet 50% of the general population requirements) or adequate intake (AI; established standard if lacking data to determine a recommended dietary allowance for a given nutrient), as appropriate. The research team met with FS during the monthly DEP meeting to discuss dietary intake.

### Statistical analysis

All data were summarized using means and standard errors or counts and frequencies, as appropriate. The 25th, 50th, and 75th percentiles were also presented for relevant variables. Qualitative feedback from recruiters and lessons learned were summarized. One-sample t tests were used to compare pre-/post-knowledge scores. One-sample Student *t* tests or Wilcoxon signed rank tests were used to compare basic training outcomes and nutrient intakes to normative values. For basic training, NRC provided normative values. Data analyses were conducted using the statistical programming language R (The R Project for Statistical Computing, Vienna, Austria), and statistical methods included regression analysis, repeated measures analysis of variance, and nonparametric testing. Significance was set at *p* < 0.05. Factors such as sex and socioeconomic status were considered for covariates.

## Results

Study enrollment was 111 FS, with 19 FS excluded from final analysis [[withdrew (*n* = 3), did not complete baseline assessment (*n* = 6), dropped from DEP (*n* = 10)]. Final analysis included 92 FS (13 females, 79 males, age = 18–28 years), of whom 23.0% identified as Asian; 9.0% as Black or African American; 40.4% as Hispanic or Latino; 1.1% as Native Hawaiian, Other Pacific Islander, or some other race; and 25.8% as White. Over half (55.0%) of the participants reported being students or having a job (part time or full time), and 92.1% indicated that they were single/never married. Most participants (85.3%) reported their highest level of education as a high school diploma or equivalent.

### Pilot engagement and outcomes

The average time in DEP was 83.5 ± 4.5 days; total and mean daily logins are presented in Table 1. More than two-thirds of the modules were completed for GMT (69.1 ± 4.1%) and nutrition (66.8 ± 4.2%). Both GMT and nutrition knowledge scores improved over the course of the DEP [GMT pre = 51.7 ± 1.6, post = 67.9 ± 2.9, *p* < 0.001, 95% confidence interval (CI) for improvement: 8.9, 20.8%; Nutrition pre = 58.1 ± 1.1, post = 68.8 ± 2.0, *p* < 0.001, 95% CI for improvement: 6.3, 14.5%]. Basic training graduation rate was 92.4% (*n* = 85; RTC attrition rates during the same period were ∼13%), with seven discharges (four preservice, one contract breach, and two medical).

**Table 1:** System usage data for 92 future sailors.

Usage type	Mean ± SE	25th percentile	50th percentile	75th percentile
Total logins	174.6 ± 17.7	60.0	131.0	225.0
Mean daily logins	2.1 ± 0.2	1.0	1.5	2.9

Higher RDC assessment scores (78 ± 5%; 95% CI: 68, 96%) were found in study FS compared with the 2021 historical average of 58% (*p* < 0.001). In addition, AFQT scores (65 ± 2%) were greater than the median score of 50 (*p* < 0.001). No differences were found in rate of advancement (18 ± 4% vs. 2021 historical average = 12%; *p* = 0.130), and DEP scores were lower for study FS compared with the 2021 historical average (actual = 3.5 ± 0.6; 2021 = 3.7, *p* < 0.001).

### Weight status, weight goals, and health beliefs

Weight status was assessed in 73 FS [female: BW = 59.6 ± 3.5 kg, body mass index (BMI) = 23.1 ± 1.0 kg/m^2^; male: BW = 76.2 ± 1.7 kg, BMI = 25.5 ± 0.5 kg/m^2^]; self-reported weight goals for 69 FS are presented in Table 2 by BMI. Of the 98 FS who completed baseline surveys, 53.8% (*n* = 45) of females and 44.7% (*n* = 38) of males indicated a weight loss goal, with 11.2 ± 0.0% of FS reporting issues with weight management. Measured BW was 1.3 ± 0.5 kg higher than self-reported realistic BW (*p* = 0.018; 95% CI: 0.2, 2.4), and weight changes in the past year were reported by 73.0 ± 0.4% of FS. BW did not change from baseline to post-DEP in 32 FS with recorded BW (*p* = 0.606).

**Table 2:** Self-reported weight goals by body mass index (BMI) classification.

BMI classification	Weight loss	Weight gain	Weight maintenance	No weight goals	Total
Underweight	0	4	1	0	5
Normal weight	6	11	9	1	27
Overweight	19	4	8	0	31
Obese	6	0	0	0	6

Values represent participant counts.

Nutrition was rated as important for performance by FS (females = 4.1 ± 1.3, males = 4.0 ± 1.0), on a scale from 1 (*not important at all*) to 5 (*extremely important*). The majority (88.8%) reported not following a specific dietary pattern (e.g., keto, Mediterranean, paleo, or vegan), and 41.8% reported using dietary supplements. Vitamin/mineral (*n* = 31) followed by protein (*n* = 20) were the most commonly consumed supplements. Most FS reported feeling “rather certain” or “very certain” that they could adhere to a healthy diet (*n* = 85) and meet exercise goals (*n* = 79).

### Energy and nutrient intake

Sixty-nine FS completed ≥1 day of food records (25.6 ± 4.4 days), with 59 FS recording dietary intake for ≥7 days. Energy and macronutrient intakes are presented in Table 3. Dietary fat intake was high (>35% of total energy intake) in 43 of 69 FS. In addition, almost all (98.4%) FS had low dietary fiber intake (8.8 ± 1.0 vs. AI = 25 g/d, *p* < 0.001 for females; 15.3 ± 1.0 vs. AI = 38 g/d, *p* < 0.001 for males) (29), while about half (31/66) had sodium intake (2248.7 ± 124.1 mg/d) higher than recommended in US Department of Agriculture Dietary Guidelines for Americans (>2,300 mg/d) (30). Suboptimal intakes of key micronutrients included calcium and vitamin D, which support healthy bone development, and antioxidants, such as vitamins C and E. Micronutrient data are reported in Table 4.

**Table 3:** Energy and macronutrient intake in future sailors.

Dietary intake	Female (*n* = 12)	Male (*n* = 54)	Recommended intake**
Energy intake (kcal/d)	1,524.4 ± 87.5*	1,789.5 ± 58.8	Female: 1,600–2,800
			Male: 1,800–3,600
Carbohydrate intake			
Absolute (g/d)	173.3 ± 15.9	177.3 ± 8.7	
Relative (g/kg)	3.2 ± 0.4*	2.4 ± 0.2	3–10
Relative (% of energy intake)	44.8 ± 2.1	39.4 ± 1.3	45–65
Protein intake			
Absolute (g/d)	76.8 ± 4.5*	99.6 ± 4.5	
Relative (g/kg)	1.3 ± 0.1	1.3 ± 0.1	1.2–2.0
Relative (% of energy intake)	20.8 ± 1.8	22.5 ± 3.9	10–35
Fat intake			
Absolute (g/d)	58.6 ± 3.9*	75.3 ± 2.7	Female: 70–100
			Male: 100–157
Relative (g/kg)	1.0 ± 1	1.0 ± 0.1	∼1
Relative (% of energy intake)	34.6 ± 1.4	38.4 ± 0.0	20–35

Values represent mean ± SE.

Macronutrient guidelines are based on the 2016 Position of the Academy of Nutrition and Dietetics, Dietitians of Canada, and the American College of Sports Medicine: Nutrition and Athletic Performance (27).

* Females differed from males (*p* < 0.05).

** Targeted caloric needs were determined by calculating resting metabolic rate using the Cunningham equation and assuming an activity factor that ranges between 1.5 and 1.9 for moderate to very heavy activity.

**Table 4:** Dietary intake for select vitamins and minerals in future sailors.

	Female (*n* = 12)			Male (*n* = 66)		
Micronutrient	Mean ± SE	RDA or AI^a^	Sufficient intake (%)^b^	Mean ± SE	RDA or AI^a^	Sufficient intake (%)^b^
Vitamin A (ug/d)^c^	797.9 ± 179.7	700	58.3	1,410.9 ± 201.6	900	67.9
Vitamin D (ug/d)	8.6 ± 2.6**	15	9.1	4.6 ± 0.6**	15	5.7
Vitamin E (mg/d)	2.9 ± 0.5**	15	0.0	4.3 ± 0.3**	15	0.0
Vitamin K (ug/d)^d^	75.3 ± 18.6	90	41.7	109.6 ± 23.8*	120	20.8
Vitamin B6 (mg/d)	0.9 ± 0.1	1.3	41.7	1.4 ± 0.1	1.3	60.4
Vitamin B12 (ug/d)	2.2 ± 0.4	2.4	36.4	3.5 ± 0.3	2.4	76.5
Folate (ug/d)	212.3 ± 46.0	400	18.2	227.0 ± 15.2	400	20.8
Vitamin C (mg/d)^d^	40.7 ± 5.9	75	25.0	59.8 ± 7.7	90	27.5
Calcium (mg/d)	324.2 ± 44.0	1,000	0	559.8 ± 34.1	1,000	18.9
Iron (mg/d)	7.8 ± 1.0	18	41.7	10.8 ± 06	8	92.5

Dietary intake was lower than estimated average requirement (EAR) or adequate intake (AI).

* *p* < 0.05.

** *p* < 0.001.

a Dietary reference intakes are a set of reference values that include recommended dietary allowance (RDA), defined as levels sufficient for ∼97% to 98% of healthy individuals; and EAR, defined as sufficient for approximately half of heathy individuals. AI for nutrients (e.g., vitamin K) is provided when those data are lacking to establish RDA and EAR.

b Sufficient intake (percentage of total participants) was determined by comparing with EAR or AI.

c Vitamin A is expressed as micrograms/day of retinol activity equivalents.

d Vitamin K and C data for male FS were not normally distributed (vitamin K median = 49.2, range = 0.4–966.2 ug/d; vitamin C median = 41.0, range = 0.2–252.7 mg/d). Thus, Wilcoxon signed rank test was used to evaluate significance.

Dietary improvements were seen in 28 FS who completed food records over a period ≥3 months (multiple days per month). Of those, 23 had inadequate fiber intake during the first month, with 16/23 (∼70%) increasing intake (grams of fiber/1,000 kcal) in the second month and 14/23 (∼61%) sustaining that increase. Twenty-two of 28 FS had high sodium intake, with 12/22 (∼55%) decreasing intake in the second month and 11/22 (∼50%) sustaining that decrease (milligrams of sodium/1,000 kcal). Eighteen of 28 FS had high fat intake (>35% in the first month), with 44% decreasing fat intake (percentage of kilocalories) in the second month and 61% maintaining that decrease (percentage of total kilocalories in the third month vs. the first month).

## Discussion

We evaluated the impact of CM+, an mHealth app, on improving the physical and behavioral readiness of FS enrolled in the DEP. Compared with historical data, CM + users had higher AFQT scores, RDC assessment scores, and graduation rates from basic training, lower DEP scores, and no difference in their rate of advancement. Approximately 67%–69% of training modules were completed by CM + users, who, consistent with the hypothesis, displayed improved knowledge of GMT and nutrition. Additionally, FS exhibited increased dietary fiber intake and reduced sodium and fat intakes throughout the DEP training period. To our knowledge, this study is the first to demonstrate enhanced physical and behavioral readiness among FS utilizing an mHealth app during the DEP.

DEP training delivered via CM+ improved military-relevant outcomes including physical fitness, GMT, RDC assessment scores, and basic training graduation rates. The RDC assessment, a physical fitness test, evaluates cardiorespiratory fitness and muscular endurance (26). Low physical fitness has been established as a modifiable risk factor for attrition and musculoskeletal injury during initial military training (31–33). Thus, the higher RDC assessment scores in the CM+ training delivery group may have contributed to the higher graduation rates observed. This group also had higher AFQT scores, a subscale of the broader ASVAB used across the Armed Forces, linked to success before and during training, in addition to selection for the Special Operations Forces (4, 34, 35), potentially pointing to increased aptitude. The lower DEP scores observed may be attributed to the potential for large gaps from when FS completed the 10-week program and their actual ship date (the DEP test is taken upon arrival to basic training). Despite this DEP scores in study participants were still above the passing minimum (3.2).

The use of CM+ during the DEP improved nutrition knowledge and dietary habits among FS. Initial self-reported dietary intakes were consistent with findings from other free-living tactical athletes (i.e., military, law enforcement, fire and rescue), reflecting adequate dietary protein, excessive dietary fat, and insufficient carbohydrate and energy intakes (36, 37). FS also reported insufficient intake of several micronutrients, including vitamins D, E, and K. Vitamin D status is an important modifiable risk factor for bone stress injuries in military recruits during training; most Navy recruits who sustain a stress fracture during basic training are either vitamin D insufficient (95%; 20 to 30 ng/mL) or deficient (82%; ≤20 ng/mL) (38). The heightened stress during basic training may accelerate bone remodeling, a process that requires adequate intakes of energy, carbohydrates, and certain micronutrients (e.g., vitamin D, calcium, vitamin K) to maintain homeostasis (39, 40). Furthermore, FS had lower intakes of calcium, a mineral important to bone health, and iron, which is important for energy metabolism, than previous investigations of male and female US soldiers (41). These results are in line with previous research reporting poor dietary habits in military personnel (e.g., meal skipping, low intake of fruits/vegetables) (42); however, FS using the CM+ app showed improvements, including reduced fat and sodium intake and increased fiber intake. mHealth apps such as CM+ have potential for monitoring and improving dietary intake and quality, helping to reduce modifiable risk factors related to injury and attrition. These improvements in diet and nutrition knowledge extend the efficacy of mHealth apps in military populations, which have primarily focused on overcoming barriers to receiving healthcare (22). Information-based interventions aimed at improving nutrition and related behaviors represent a promising direction for mHealth technology.

This study had several limitations. The frequency of meal reporting was generally low, and potential underreporting could have contributed to the inadequate intakes observed in this study. The days participants recorded their food intakes were not standardized and may not have reflected habitual intake. Self-report of other data (e.g., physical training, questionnaires) may have also added error and/or bias to results. While dietary intake differences were reported to visualize trends between sexes, we were underpowered to assess any other group differences. Another constraint was the lack of a control comparison group, which has been used in other mHealth interventions (23). Lastly, this study was also limited by the small sample size and the restricted recruitment location. To obtain adequately powered results, future research should include a larger and more geographically diverse participant pool. Investigation into the use of CM+ should extend to other military groups, such as active duty Navy personnel and service members from different branches, to provide health behaviors education and job-specific training. By monitoring, evaluating, and integrating data related to the unique physical and mental demands of these groups, the recruitment process could be refined, leading to enhanced warfighter health and performance.

In conclusion, the evaluation of the mHealth app CM+ demonstrated its effectiveness in improving physical and behavioral readiness of FS enrolled in the DEP. CM+ users achieved superior results in military-relevant metrics, including enhanced physical performance and higher basic training graduation rates. Participants also demonstrated increased knowledge of GMT and nutrition, along with improved dietary habits. This study, to our knowledge, is the first to demonstrate improved physical and behavioral readiness outcomes among FS equipped with an mHealth app while enrolled in the DEP. This study provides evidence that mHealth interventions are effective tools for enhancing readiness in FS, equipping them for successful entry into military service.

## Data Availability

The datasets presented in this article are not readily available because data sharing is strictly regulated by our institution. Requests to access the datasets should be directed to lynn.a.kam.civ@health.mil.

## Author Disclaimer

I am a military service member or employee of the US Government. This work was prepared as part of my official duties. Title 17, U.S.C. §105 provides that copyright protection under this title is not available for any work of the US Government. Title 17, U.S.C. §101 defines a US Government work as work prepared by a military service member or employee of the US Government as part of that person's official duties. Report No. 25–34 was supported by the Office of the Chief of Naval Operations (OPNAV N17) under work unit number no. N2011. The views expressed in this article are those of the authors and do not necessarily reflect the official policy or position of the Department of the Navy, Department of Defense, nor the US Government. The study protocol was approved by the Naval Health Research Center Institutional Review Board in compliance with all applicable federal regulations governing the protection of human subjects. Research data were derived from approved Naval Health Research Center Institutional Review Board protocol number NHRC.2021.0010.
